# Bacterial composition and physicochemical characteristics of sorghum based on environmental factors in different regions of China

**DOI:** 10.3389/fmicb.2024.1422471

**Published:** 2024-06-28

**Authors:** Peiyun Xie, Mingbo Shao, Xiaofeng Deng, Yan Ren, Manjing Chen, Yuwen Jiang, Jiaqi Shen

**Affiliations:** ^1^Guizhou Light Industry Technical College, Guiyang, China; ^2^Institute of Upland Food Crops, Guizhou Academy of Agricultural Sciences, Guiyang, China

**Keywords:** Jiang-flavored baijiu, sorghum, physicochemical indexes, bacterial community, environmental factors

## Abstract

The fermentation process for Jiang-flavored baijiu using sorghum as the raw material involves a variety of microorganisms. However, the specific physicochemical characteristics of sorghum and microbial composition on its surface have not been fully elucidated. We aimed to perform a comprehensive comparative analysis of the variations in physicochemical properties and surface microflora in waxy sorghum samples from three prominent production regions in China (Renhuai, Jinsha, and Duyun). Multivariate statistical assessments were conducted that incorporated local soil and climate variables. The results showed that Cyanobacteria, unclassified bacteria, Proteobacteria, Firmicutes, and Bacteroidota were the dominant bacteria in these regions. These bacteria were associated with ethyl acetate, ethyl caprylate, ethyl lactate, and butyl groups, which synergistically produce flavorful compounds. The surface bacterial communities were affected by soil total phosphorus, altitude, diurnal temperature range, monthly mean temperature, precipitation, and effective accumulated temperature. The findings of this study provide a new perspective on microorganisms related to Jiang-flavored baijiu and can help establish a reference for the stability of liquor quality.

## 1 Introduction

Jiang-flavored baijiu is one of the world’s highest-selling distilled alcoholic spirits made from waxy sorghum (*Sorghum bicolor* L. Moench) as the main raw material (Niu et al., 2022; Wang, 2022). It is produced by solid-state fermentation and distillation, resulting in a strong soy sauce aroma and a long-lasting fragrance (Yan, 2019; Gao et al., 2024). Regional variations in the sorghum and baijiu quality characteristics are critical features of the perceived product identity, with significant consequences for consumer preference and economic appreciation (Liu et al., 2023). These variations may be associated with local climatic and soil conditions that play important roles in sorghum quality and microbiology (Niu et al., 2022).

The liquor quality depends on the physicochemical properties of the sorghum (Liu et al., 2021a). Starch, protein, fat, and other components of sorghum can not only be used as substrates that provide energy for microorganisms during the fermentation process but can also generate many flavor compounds or participate in subsequent metabolic reactions as flavor precursors (Li E. et al., 2021; Li H. et al., 2021). Numerous studies have reported differences in the physicochemical indices and regional characteristics. It is generally agreed that southern Chinese sorghum has a higher content of amylopectin, fat, and tannins than sorghum from northern China. In addition, differences in the amino acid metabolism, phenylpropanoid biosynthesis and flavonoid biosynthesis pathways result in a higher production of acid ester flavoring substances, making southern Chinese sorghum more suitable for brewing Jiang-flavored baijiu with optimal flavor and quality than sorghum from the north (Zhang et al., 2022). However, the same variety of sorghum grown in different areas may also be affected by environmental factors, such as soil and climate, resulting in different physicochemical properties.

The brewing process of Jiang-flavored baijiu is a complex natural multispecies solid fermentation process in which bacteria, actinomycetes, yeasts, and molds work together to establish a complex, dynamic, and balanced microbial ecosystem (Tu et al., 2022; Xu et al., 2022). Theoretically, many microorganisms inhabiting the sorghum surface cannot survive at high temperatures but their metabolic activities can have long-term effects (Bokulich et al., 2014; Gao et al., 2019). Sorghum is one of the main sources of bacteria during the fermentation process. During the production of Jiang-flavored baijiu, grain wetting, steaming, cooling, mixing, and bacterial cultivation are all conducted in the same semi-open plant so that the microorganisms on the surface of the sorghum raw material can become airborne and integrate into the brewing environment (Dai et al., 2020; Fan and Xu, 2023). In addition, during the grain-turning process, sorghum may be exposed to uneven heat, which can result in the survival of some microorganisms in the form of spores that can survive and enter the subsequent fermentation processes. The structure of the microbial communities on crop surfaces is often influenced by environmental factors. Research has shown that habitat heterogeneity shapes the distribution of fungi and bacteria in brewing environments (Ren et al., 2023; Yuan et al., 2023), while climatic conditions and geographic factors may play a role in controlling microbial community composition (Ding et al., 2021). However, it is unclear whether there is a non-random geographical distribution pattern of microorganisms on the surface of waxy sorghum and understanding their distribution patterns and mechanisms requires further exploration.

Guizhou Province is the core production area for Jiang-flavored baijiu owing to its unique geography and climate. Improvements in the efficiency of the liquor industry have effectively stimulated demand for brewing sorghum (Liu et al., 2021b). Starting in 2020, Guizhou Province prioritized the waxy sorghum planting industry as a key commercial opportunity, with the authorities in Guizhou establishing sorghum planting demonstration sites in addition to Renhuai (e.g., a prominent production region) and gradually forming suitable production areas in the north, southwest, and south of Guizhou. Although the fermentation characteristics of waxy sorghum production in various regions have been reported, there is a lack of information on the physicochemical properties and microbial community composition on the surface of the same variety sourced from different locations. Therefore, the aim of this study was to analyze the physicochemical properties and obtain information on the structure of the microbial communities and dominant functional genes in sorghum from production regions in Renhuai, Jinsha, and Duyun using high-throughput sequencing. To explore the factors that influence surface microorganisms, we collected soil from different production areas and downloaded the relevant meteorological data. This study provides information that supports the improvement of baijiu enterprise product stability and competitiveness through differential development. This study also provides an important scientific basis for evaluating microbial resources for brewing.

## 2 Materials and methods

### 2.1 Sample source

**Figure 1:**
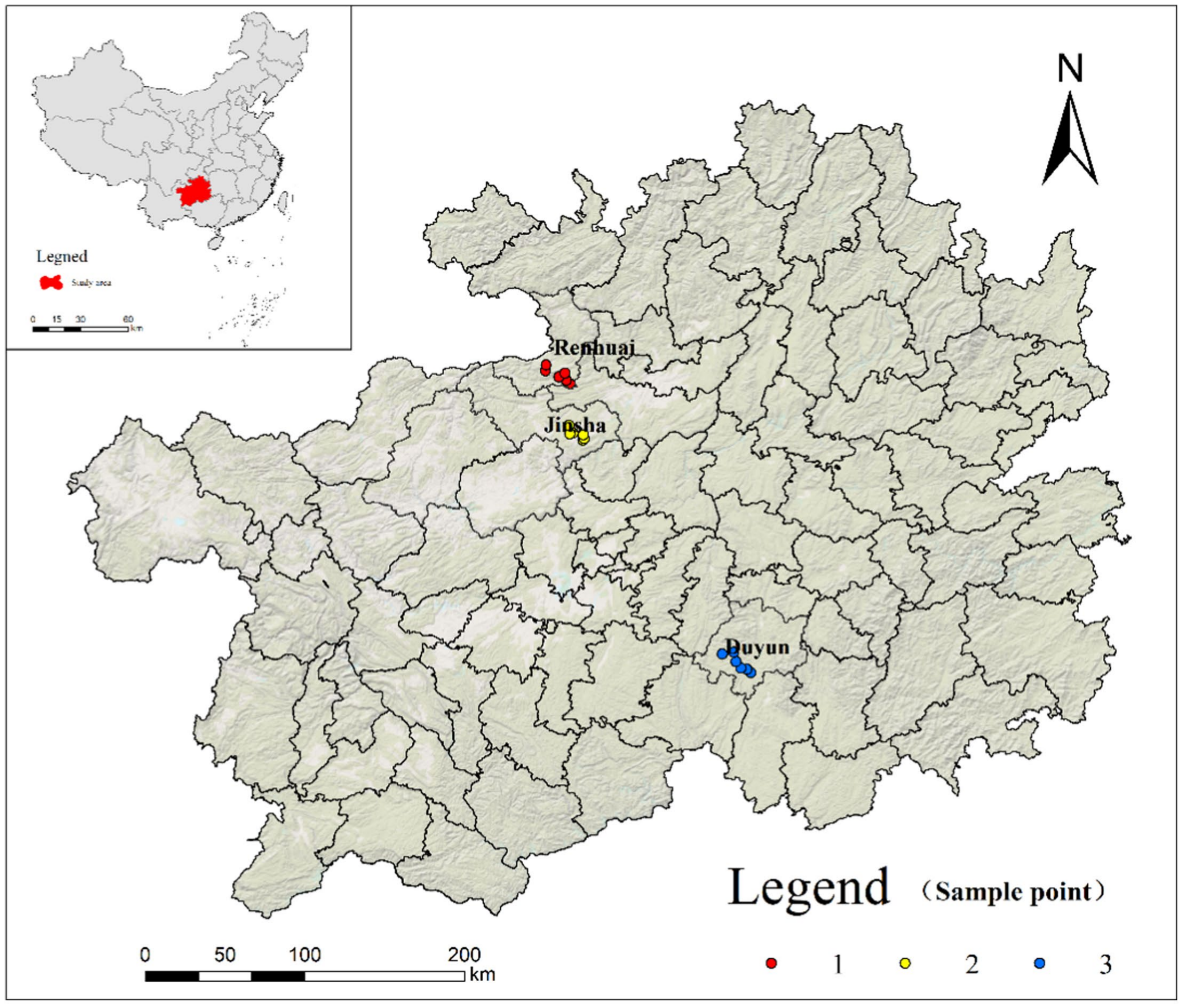
Geographic locations of sampling sites.

The sorghum variety ‘Hongliangfeng No. 1’ [registration number: GPD Sorghum (2017) 520029] was used in this study. The sorghum was sourced from a factory that obtained waxy sorghum from three supply bases: Renhuai (RH), Duyun (DY), and Jinsha (JS). All three bases are in the key waxy sorghum industrial belt in Guizhou Province, China (Figure 1). In August 2022, six experimental sample plots were randomly selected at each base and the latitude, longitude, and elevation of the sample plots were recorded using a handheld GPS.

Soil samples were collected alongside the sorghum samples. Sorghum batches from all three supply bases were harvested and numbered to indicate their origin (e.g., RH sorghum, DY sorghum, or JS sorghum). Fermented grain samples were collected from the same distillery and workshop. Sorghum from the three regions was first cooked and fermented in piles for 48 h. Then, we collected three portions from the top, middle, and bottom layers of each pile and mixed them well. Finally, we analyzed the samples to determine their volatile compound content.

### 2.2 Sample collection

Sorghum samples were collected from five randomly selected healthy sorghum plants with the same length from each experimental plot. Sorghum plants of a uniform size and similar color without obvious pests or diseases were selected, placed in sterilized bags and transported to the laboratory via a cold chain at 4°C. Sorghum collected from the nine sample plots was thoroughly mixed and divided into three portions for testing. Each sample was split in half, with one half used for sorghum grain quality analysis and the other half used for high-throughput sequencing after extracting the surface bacterial DNA. Soil samples were collected from the inter-root soil (0–30 cm) of each mature sorghum kernel. Debris, such as litter and gravel, were removed and the soil samples were passed through a 2-mm sieve. The samples were then brought back to the laboratory to determine their chemical properties. Fermented grain samples were taken from the top, middle, and bottom of three fermented grain piles (20 cm); six sampling points and three replicates of the samples were collected, placed in Ziplock bags and transported to the laboratory via a cold chain at 4°C to determine the volatile components.

### 2.3 Climate data

Sorghum at the three bases (RH, DY, and JS) was planted at the end of March and harvested at the beginning of September 2022. Therefore, climate observation data from March to September 2022 were selected. These data included the average monthly temperature, effective accumulated temperature, diurnal temperature variation, precipitation, and relative humidity, which were obtained from the daily value dataset of surface climate data provided by the China Meteorological Science Data Sharing Service Network.

### 2.4 Chemical analysis

The starch content in sorghum was measured as previously described (McCleary et al., 1994). Amylose and amylopectin content were determined using the ISO 6647-2-2007, protein content was determined using the ISO 11085:2015, fat content was determined using the ISO 20483:2013 standard and tannin content was determined using the ISO 9648:1988.

Soil samples were analyzed using agricultural chemical analysis of soil protocols (Bao, 2000) for the indicators, pH, organic matter, total nitrogen, total phosphorus, total potassium and rapidly available nitrogen, phosphorus, and potassium.

### 2.5 Volatile compound analysis

Volatile compounds were analyzed using headspace solid phase microextraction. The pretreatment method was used as previously described (Ren et al., 2023) with some modification. We added 2.00 g fermented grains into a 20 mL headspace vial, to which 5 mL saturated NaCl solution was added. Then, 6 μL 4-octanol (0.5 mg/mL) was added as an internal standard and the sample was sonicated for 10 min at 90 W. The volatile compounds were extracted using a 50/30 μm DVB/CAR/PDMS fiber (Supelco, Bellefonte, PA, USA) and analyzed using HS-SPME-GC–MS (TSQ 8000 Evo, Trace MS/GC; Thermo Fisher Scientific, Waltham, MA, USA) equipped with a TG-5MS column (30 m × 0.25 mm × 0.25 μm; J&W Scientific, Folsom, CA, USA) and a flame ionization detector. The gas chromatography–mass spectrometry had a starting temperature of 40°C held for 3 min, which was then increased to 100°C at a rate of 2°C/min and held at 100°C for 5 min before it was increased to 150°C at a rate of 2°C/min and held at 150°C for 2 min, and finally increased to 230°C at a rate of 10°C/min and held at 230°C for 5 min. Mass spectra were generated in electron ionization mode at 70 eV. The full-scan mode ranged from 28 to 500 amu. Quantitative analysis of the volatile substances was performed by matching the standard mass spectra with the NIST11.al spectral database (Agilent Technologies, Inc., Santa Clara, CA, USA).

### 2.6 High-throughput sequencing analysis

Sterile water (450 mL) was added to 50 g sorghum and the mixture was then sealed before being shaken at a constant temperature for 30 min. A 0.22 μm filter membrane was used to extract the bacterial suspension and the membrane was sent to Beijing Biomarker Biotechnology Company Limited at 4°C for sequencing. The 338F (5′-ACTCCTACGGGAGGCAGCAG3′) and 806R (5′-GGACTACHVGGGTWTCTAAT-3′) primers were used to amplify the 16S rRNA gene of the bacterial V3–V4 variable region. The amplification procedure was as follows: 94°C pre-denaturation for 4 min, 30 cycles of 94°C denaturation for 60 s, 53°C annealing for 60 s, 72°C extension for 60 s and 72°C extension for 10 min. The reaction was composed of 10 × 2.5 μL FastPfu buffer, 0.25 μL 2.5 mmol/L dNTPs, 0.4 μL primers (5 μmol/L), 0.4 μL FastPfu polymerase and 10 ng DNA template. The PCR products were detected on 2% (w/v) agarose gels and the size of the paired-end sequence was no less than 550 base pairs. The PCR products were analyzed using the Illumina MiSeq platform (San Diego, CA, USA). Raw data were filtered to remove joints and low-quality sequences through Quantitative Insights into Microbial Ecology (QIIME2 v2020.6). Operational taxonomic units (OTUs) were clustered with a threshold of 97% sequence similarity using UPARSE software (version 10) and diversity indices, including Shannon, Simpson, Chao 1, and phylogenetic diversity (PD) tree indices, were calculated using QIIME2 2020.6 (Wang, 2022). The metabolic function prediction tool for the flora was passed through the Phylogenetic Investigation of Communities by Reconstruction of Unobserved States (PICRUSt). The 16S rRNA gene sequences from the samples were used for metabolic function prediction with the Kyoto Encyclopedia of Genes and Genomes (KEGG) database (Louca et al., 2016).

### 2.7 Statistical analysis

One-way analysis of variance (ANOVA) was used to evaluate the effects of the soil chemical properties, bacterial community diversity, and relative abundance of predicted functional genes in different production areas. Pearson’s correlation was used to analyze the relationship between bacterial community composition and flavor matter before the significance of the results was corrected for the false discovery rate (FDR) by p-adjustment. These analyses were performed using Origin 2021 software^1^. Key environmental factors affecting the bacterial community structure were assessed by redundancy analysis (RDA) using Canoco 5.0^2^.

## 3 Results

### 3.1 Differences in the physicochemical properties of sorghums

**Figure 2:**
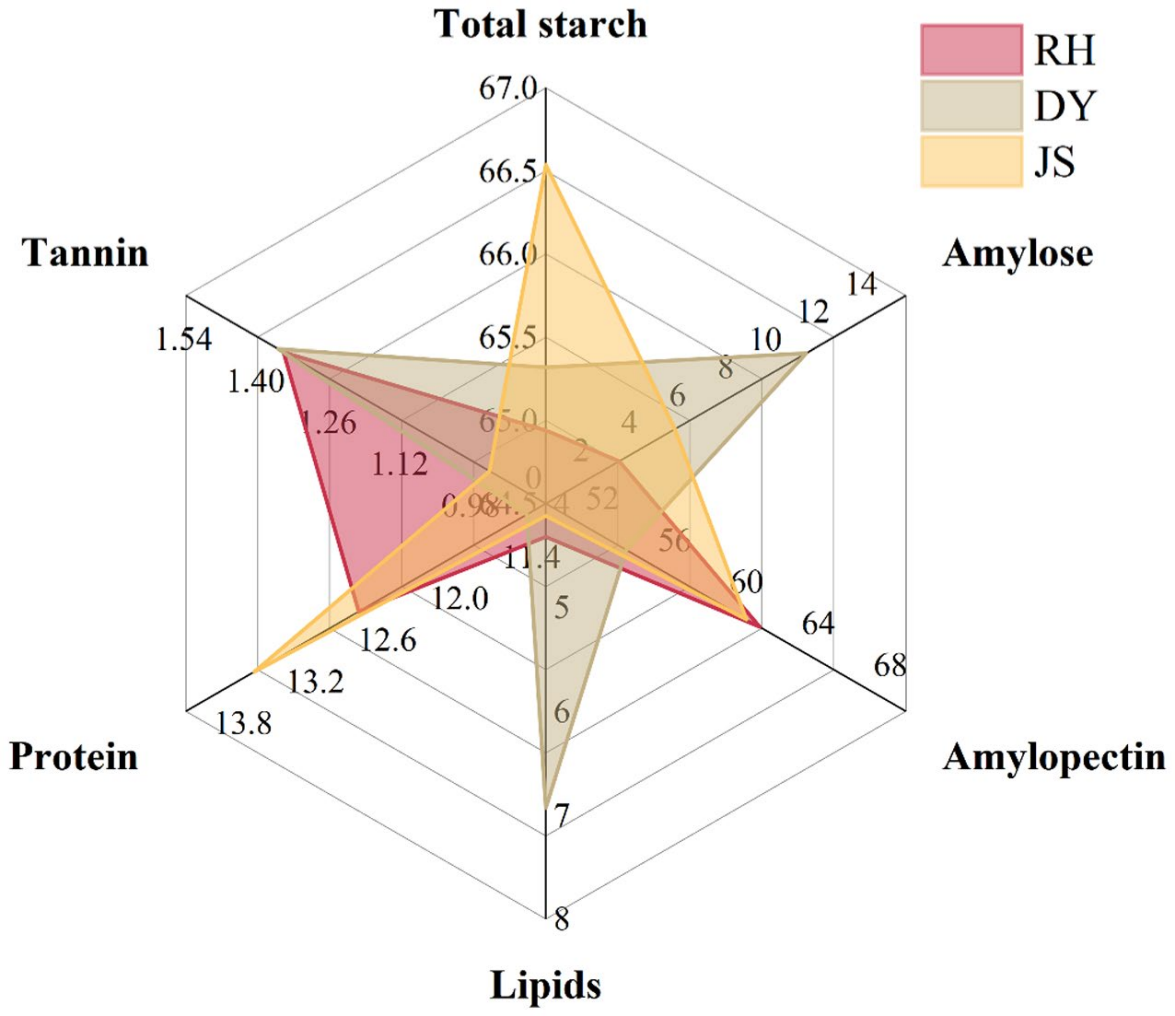
Characteristics of physicochemical quality of sorghum grain in different production areas. RH, Renhuai; JS, Jinsha; DY, Duyun.

We found that JS sorghums exhibited the highest total starch content at 66.54%, whereas RH sorghums had the lowest total starch content (64.94%) but the highest amylopectin content (61.88%), indicating significant differences among the three producing areas, while the content of amylopectin in DY was significantly lower than that in other areas (54.46%) (Figure 2 and Supplementary Table S1). Across all the regions, amylopectin accounted for over 96% of the total starch content. A comparison of the tannin, protein, and fat content across the three regions indicated that the order of their content levels was DY > RH > JS, which underscored the variations in the physicochemical characteristics of sorghum sourced from the three production regions.

### 3.2 Comparison of bacterial communities

**Figure 3:**
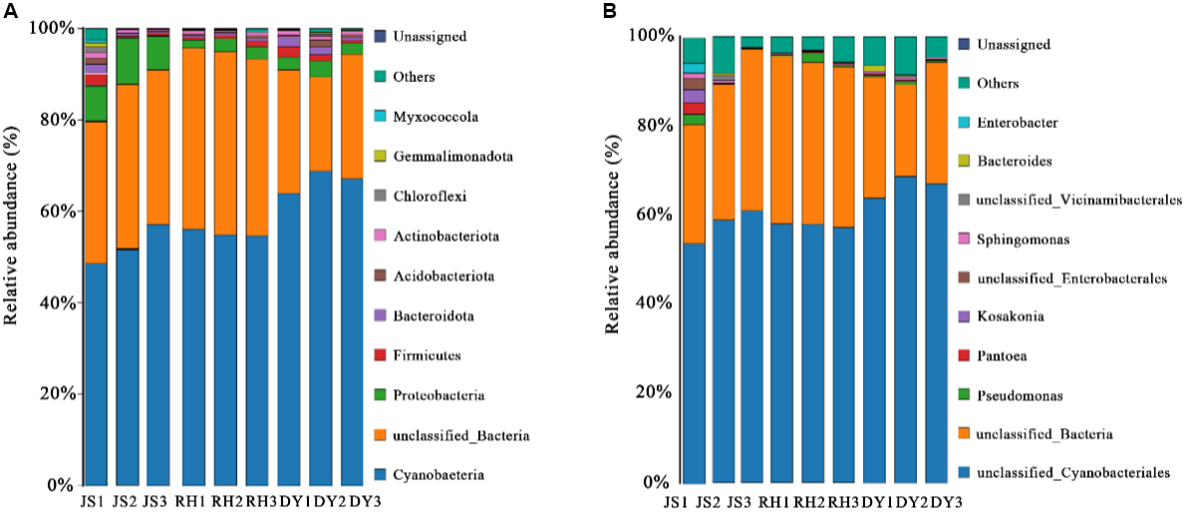
Microbial community composition among sorghum samples from the three production areas. Diagrams for bacteria **(A)** phylum and **(B)** genus abundance. RH, Renhuai; JS, Jinsha; DY, Duyun.

**Figure 4:**
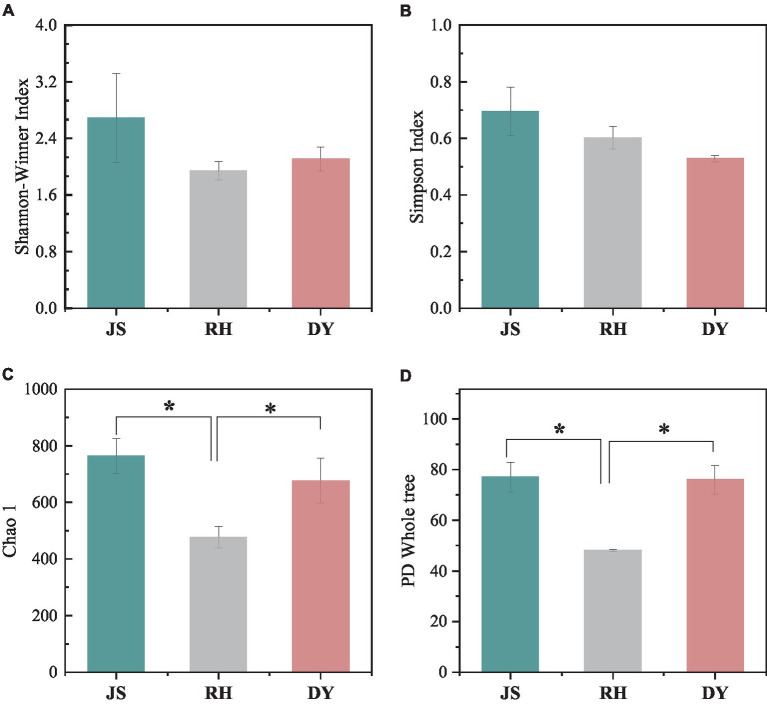
Species alpha diversity among the three regional sorghum samples. **(A)** Shannon–Winner index; **(B)** Simpson index; **(C)** Chao 1 index; **(D)** PD tree index. RH, Renhuai; JS, Jinsha; DY, Duyun.

We next examined the variations in sorghum-surface bacterial community composition across the three distinct production regions at both the phylum and genus levels. The predominant bacterial phyla identified were Cyanobacteria, unclassified bacteria, Proteobacteria, Firmicutes, and Bacteroidota (Figure 3A). Each exhibited a relative abundance that exceeded 1%, with the Cyanobacteria phylum more abundant in the DY samples (66.29%) than in the RH samples (45.77%). This difference in phyla composition represented a statistically significant difference. The percentage of Proteobacteria in the samples was highest in RH samples at 19.20%, when compared with that in the JS (15.23%) and DY (2.83%) samples. The abundance of Acidobacteria was 1.15% in JS, 0.74% in DY, and 0.39% RH samples. At the bacterial genus level, the dominant bacteria included unclassified Cyanobacteriales, unclassified bacteria, Pseudomonas, Pantoea and Kosakonia (Figure 3B). The relative abundance of Pseudomonas in the RH sample was significantly higher (17.49%) than that in JS (2.52%) or DY (0.44%). The relative abundance of Pantoea (4.36%) and Kosakonia (2.49%) was significantly higher in the JS production area than in the other two regions. However, no statistically significant differences were observed in the Shannon–Winner and Simpson diversity indices across the three production areas (Figure 4). Furthermore, the Chao 1 and PD tree indices showed no significant variation between the DY and JS samples, whereas both indices were notably higher in the RH production area.

**Figure 5:**
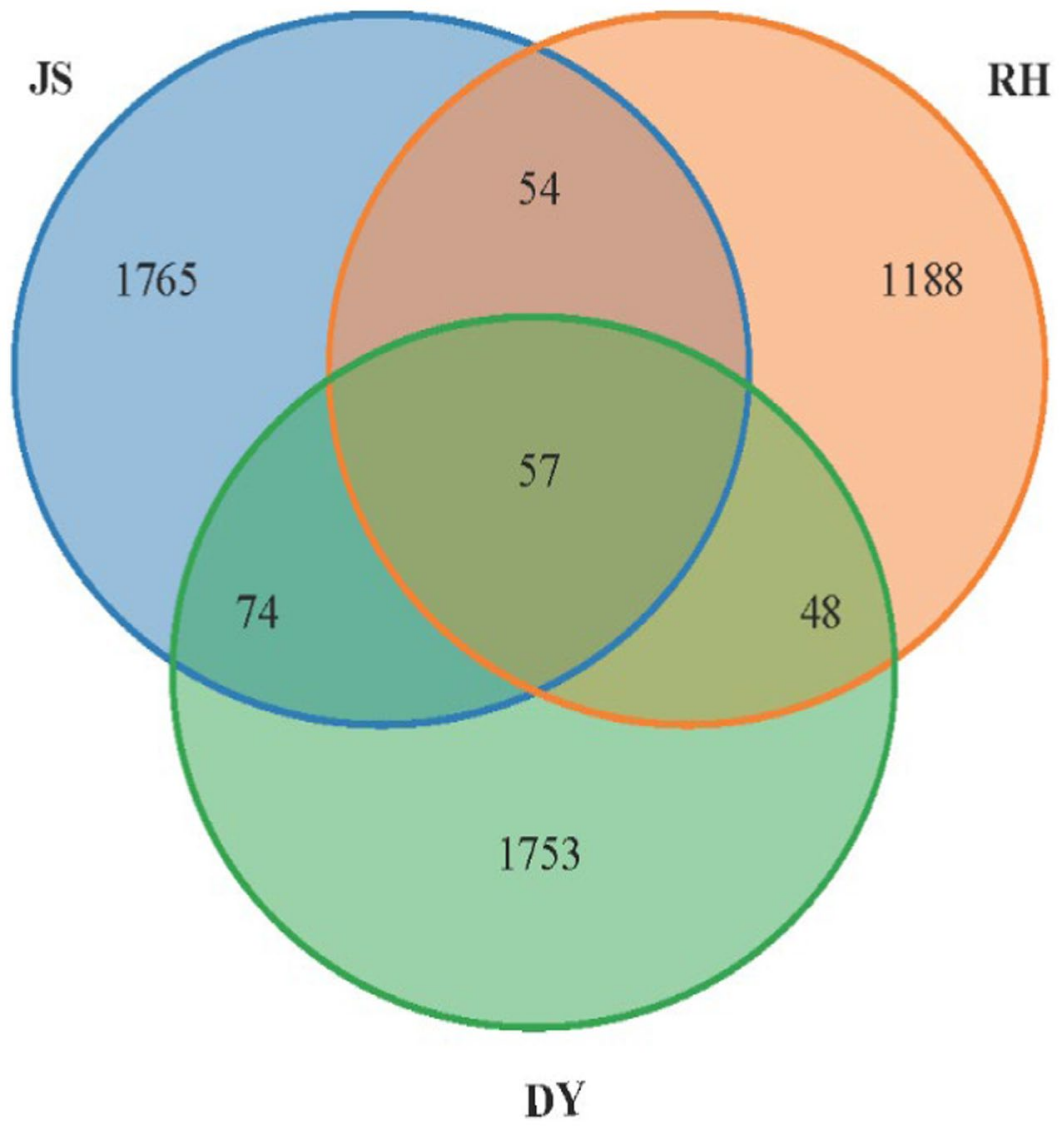
Venn diagram of the OTUs. RH, Renhuai; JS, Jinsha; DY, Duyun.

Venn diagrams were used to depict the distribution of shared and unique OTUs between samples (Figure 5). In the present study, 4,939 bacterial OTUs were identified across the samples from the three different regions, with 1950 OTUs in JS, 1347 in RH and 1923 in DY. The proportion of 57 shared OTUs accounted for only 1.15% of the overall OTUs. Conversely, the number of OTUs exclusive to each origin far exceeded the number of shared OTUs, indicating substantial differences in the bacterial community composition among the three sorghum production regions.

### 3.3 Environmental differences among sorghum production regions

Throughout the growing season, the mean monthly temperature was highest in RH, followed by DY and then JS. This pattern was primarily driven by the topographical differences; as the RH area is situated at a lower elevation it experiences progressively higher temperatures, particularly in the month of June. The diurnal temperature variation significantly impacts crop quality, with higher variations leading to increased levels of photosynthetic products, such as sucrose and starch (Tu et al., 2022). The diurnal temperature variation of the regions was in the descending order of DY > JS > RH during the growing seasons (Supplementary Figure S2B). However, the soil physicochemical properties, including pH and total nitrogen and phosphorus content, did not show significant differences among the three production areas (*p* > 0.05).

**Figure 6:**
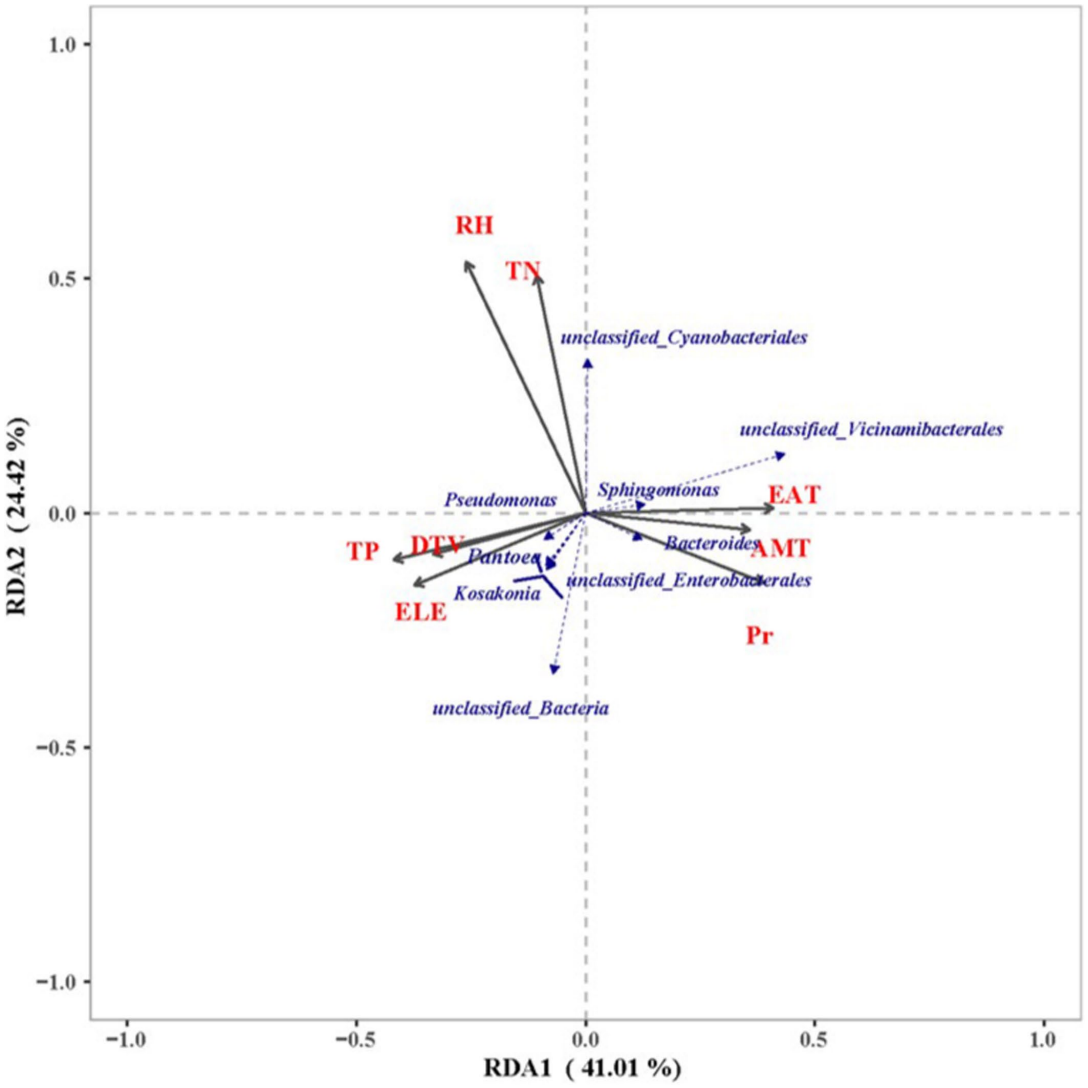
Ordination biplot showing the first two-axis results from redundancy analysis (RDA) of microbial communities. TN, total nitrogen; TP, total phosphorus; AMT, average monthly temperature; EAT, effective accumulated temperature; DTV, diurnal temperature variation; Pr, precipitation; RH, relative humidity; ELE, elevation.

When comparing the organic matter content (Supplementary Figure S3) of the various production areas, the RH production area exhibited the highest content, followed by the DY production area. The levels of total phosphorus and rapidly available nitrogen and phosphorus were significantly higher in the DY production area than in the RH and JS production areas (*p* < 0.05). Analysis of the rapidly available potassium content indicated a descending ranking of JS > DY > RH, with a highly significant difference observed among the three production areas (*p* < 0.001). The analysis of bacterial taxa and their abundance revealed a first axis gradient of 0.785 that was below the threshold of 3.0, suggesting that bacterial taxa and environmental factors were conducive to RDA. Subsequent RDA demonstrated that the cumulative explanatory rates of the first and second ordination axes were 41.01 and 24.42%, respectively, indicating statistical significance (Figure 6).

The impact of environmental factors on bacterial communities was assessed and ranked in descending order as follows: ELE > EAT > AMT > RH > Pr > DTV > TP > TN. Stronger correlations were observed between Pseudomonas, Pantoea and Kosakonia and soil TP, ELE, and DTV at smaller angles. The unclassified Cyanobacteriales showed stronger correlations with soil TD and relative humidity when the angles were smaller. Notably, a strong correlation was observed between unclassified Cyanobacteriales and soil TD, ELE, and DTV, with small correlation angles, suggesting a significant increase in their abundance with higher levels of these soil properties. Conversely, Bacteroides, unclassified Vicinamibacterales and Sphingomonas were primarily influenced by the monthly mean temperature, precipitation, and effective accumulative temperature.

### 3.4 Functional gene prediction

**Figure 7:**
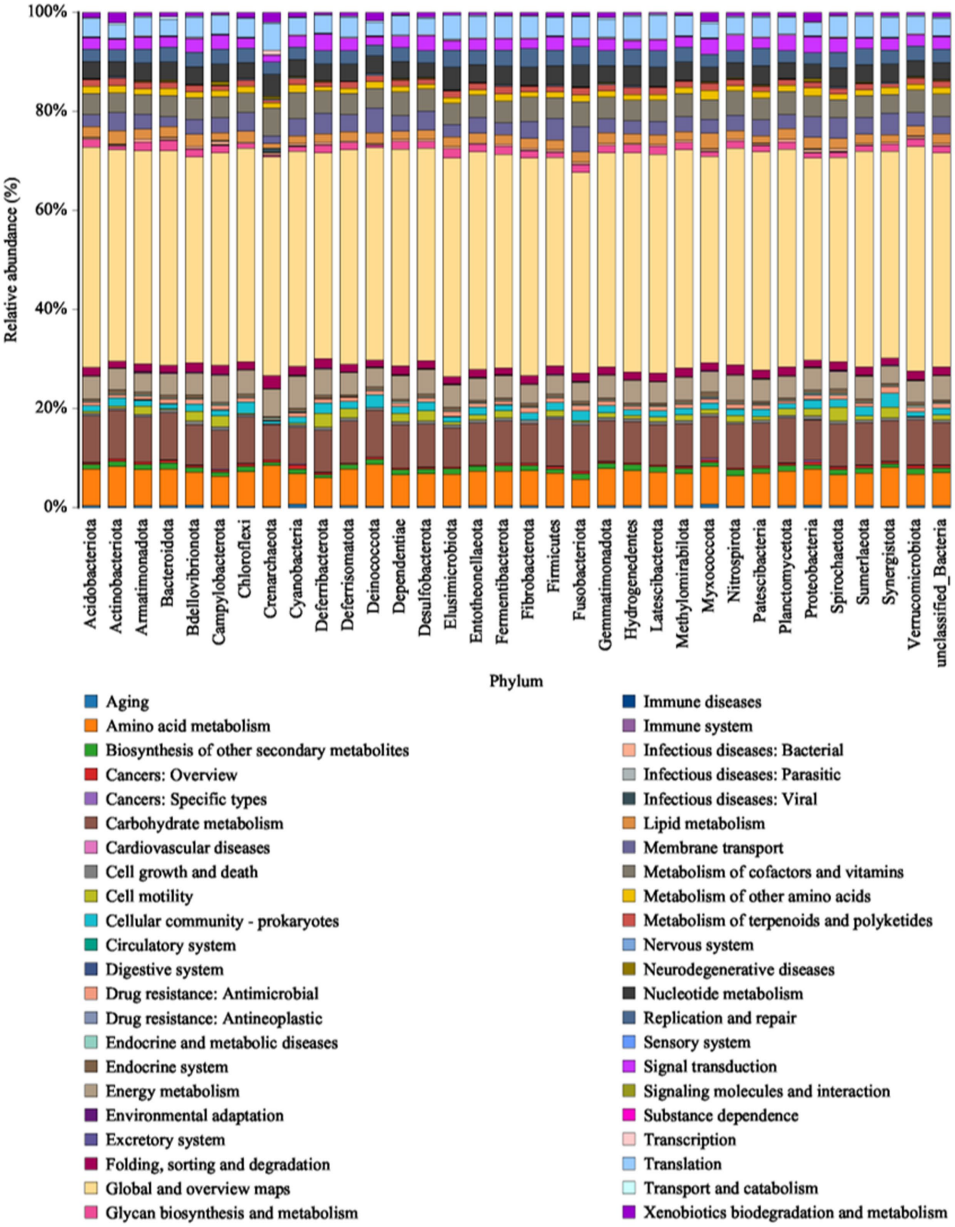
Histogram of the KEGG metabolic pathways associated with microbial communities.

PICRUSt compares microbial community abundance with a marker gene sequencing profile database to predict bacterial community function. By using high-throughput sequencing technology and comparing it with the KEGG database (Figure 7), we observed that the bacterial genes were associated with six types of metabolic pathways at the primary functional level: cellular processes (2.65%), environmental information processing (6.83%), genetic information processing (7.06%), metabolism (78.39%), human diseases (3.15%), and organismal systems (1.92%). Metabolism was the predominant functional component (Supplementary Figure S2) while at the secondary functional level, 41 categories of metabolic pathways were identified, 16 of which exhibited relative abundances greater than 1% (Supplementary Figure S3). Notably, global and overview maps, carbohydrate metabolism, amino acid metabolism, energy metabolism and the metabolism of cofactors and vitamins emerged as the major metabolic functions in bacterial communities. However, there was no significant difference in the number of functional genes within the bacterial communities across the different sorghum production regions at either the primary or secondary functional levels, indicating that various strains of sorghum-surface bacteria have unique functions irrespective of regional differences. The bacterial communities at the phylum level, which are closely related to secondary functions, are shown in Figure 7. Proteobacteria, Bacteroidota, Firmicutes, Acidobacteriota, Actinobacteriota, Chloroflexi, Myxococci, and Gemmatimonadota had higher relative abundances in metabolic pathways, such as carbohydrate, amino acid, and energy metabolism, as well as the metabolism of cofactors and vitamins. Thus, these phyla may be important in the production of the alcohols, aldehydes, acids, and esters that act as flavoring substances in baijiu. The bacterial communities on the sorghum surface are involved in processes, such as saccharification and fermentation during the brewing process and may be key players in metabolite production.

### 3.5 Correlation of flavor substances with microorganisms

**Figure 8:**
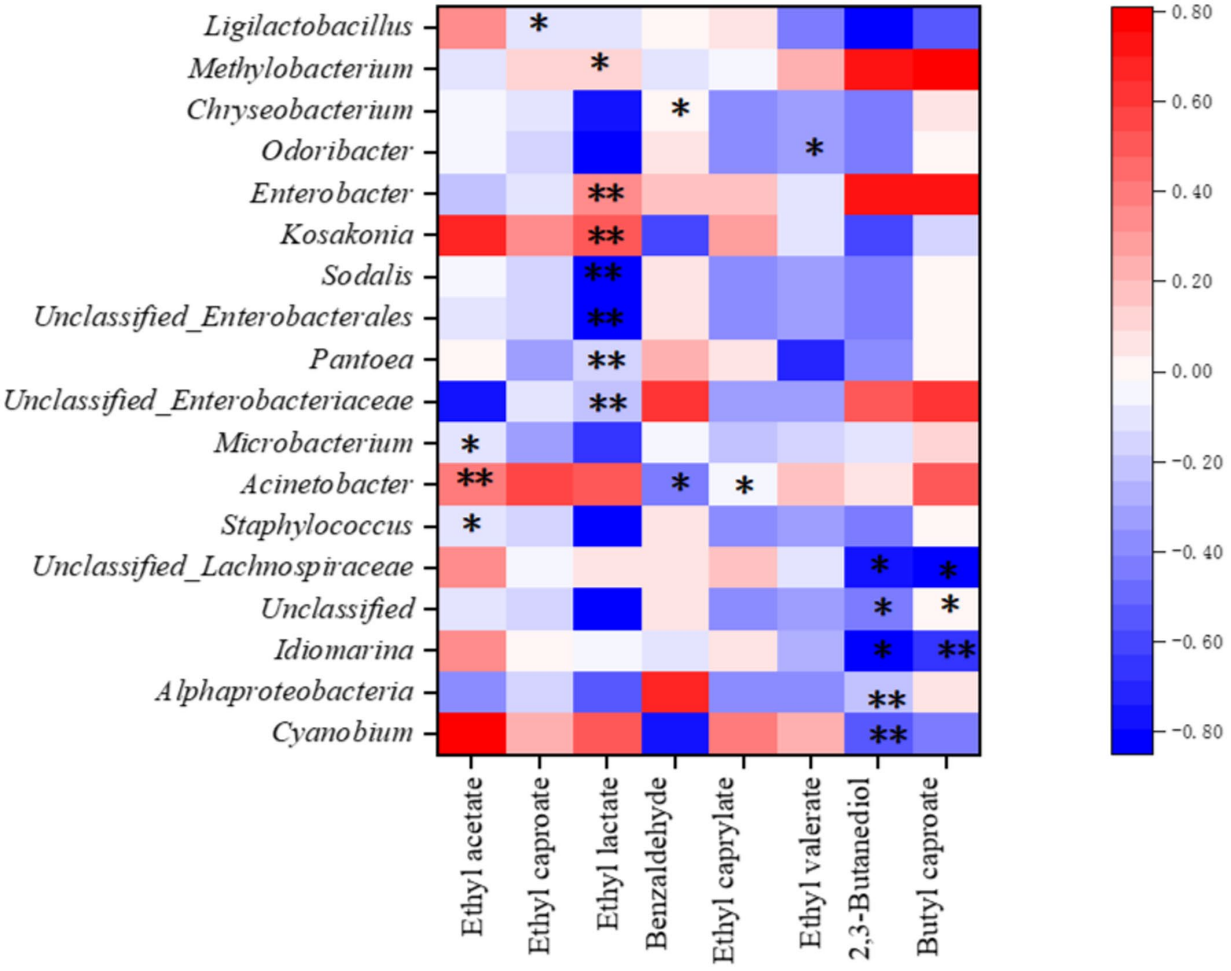
Correlation among the microbial community and baijiu flavor substances. * Significantly different (*p* < 0.05); ** highly significantly different (*p* < 0.01).

Correlation analysis between bacterial communities and flavor compounds indicated a strong positive correlation between ethyl acetate and Acinetobacter (*p* < 0.01) and significant negative correlations between ethyl acetate and Staphylococcus and Microbacterium (*p* < 0.05) (Figure 8). Furthermore, Acinetobacter was significantly and negatively correlated with benzaldehyde and ethyl caprylate (*p* < 0.05), while ethyl lactate synthesis was significantly positively correlated (*p* < 0.05) with Kosakonia, Enterobacter, and Methylobacterium and significantly negatively correlated (*p* < 0.05) with Sodalis and Pantoea. Butyl caproate showed a significant negative correlation (*p* < 0.05) with Idiomarina, whereas unclassified Lachnospiraceae showed a significant negative correlation with these bacteria (*p* < 0.05). This correlation pattern was very similar to the production of 2,3-butanediol, although it was negatively correlated with Alphaproteobacteria and Cyanobium. These results showed that there is a correlation between these flavor compounds and the diversity of the bacterial community on the sorghum surface.

## 4 Discussion

Variations in the flavor profile of Jiang-flavored baijiu within a single batch are attributed to differences in the raw materials, with particular emphasis on the impact of the chemical properties of sorghum. Numerous studies have investigated the influence of factors such as starch type, starch content, protein content and tannin content on the flavor profiles of baijiu (Li et al., 2021; Ucella-Filho et al., 2022; Wang et al., 2023). Starch, as the primary carbon source for brewing microorganisms, plays a crucial role in the production of ethanol and generation of flavor precursors (Niu et al., 2022; Pang et al., 2023). The waxy sorghum used for brewing must possess a total starch content within the range of 50–75%, as stipulated by the brewing guidelines for Jiang-flavored baijiu (Cheng et al., 2022). Notably, many factors influence baijiu yield, with a positive correlation between the total starch content and yield (Ren et al., 2023). Our investigation revealed that the total starch content of the Hongliangfeng No. 1 waxy sorghum complied with the brewing specifications irrespective of where it was produced; however, variations in the chemical parameters among the sorghum obtained from the different production regions were observed. The JS sorghum exhibited elevated levels of total starch, RH sorghum had a notable abundance of amylopectin and DY sorghum had the highest concentrations of tannin, protein, and fat of the three varieties. The fermentation of Jiang-flavored baijiu requires nine rounds of stewing, eight rounds of fermentation, and seven rounds of extraction. Aroma substances are gradually released into fermented grains during the decomposition process (Li et al., 2021; Xu et al., 2022). Therefore, the amylopectin content of sorghum is a crucial factor in determining the quality of Jiang-flavored baijiu. Proteins, fats, and tannins are also important components in baijiu production; however, the inverse relationship between tannin content and yield can be attributed to the detrimental effect of excessive tannins on microbial activity, which thereby hinders fermentation (Ucella-Filho et al., 2022). The flavor of sorghum-brewed Jiang-flavored baijiu from different production areas is influenced by the chemical properties of the raw materials of the original plant.

Although many articles have been published on the effects of different sorghum varieties on baijiu flavor (Zhang et al., 2022; Liu M. et al., 2023; Liu M.-K. et al., 2023), little attention has been paid to how differences in the microbiota on the sorghum surface affects baijiu flavor. Baijiu production involves both positive and negative interactions among bacteria, yeasts, and molds, with the final metabolic products of the continuous adaptation and domestication of microorganisms in anaerobic or aerobic fermentation environments functioning as flavor substances (Xu et al., 2022; Pan et al., 2023). Our analysis of microorganisms on the sorghum surface showed that Cyanobacteria, unclassified bacteria, Proteobacteria, Firmicutes, and Bacteroidota were the dominant bacterial phyla. Firmicutes, Proteobacteria, and Cyanobacteria are the dominant bacterial phyla in the subsequent stacking fermentation of Jiang-flavored baijiu and have a positive effect on the production of both ethanol and other flavor components (Yan, 2019; Wang, 2022). Exploring the sources of these dominant flora has been a popular research topic in recent years, which has highlighted that microorganisms for stack fermentation may originate from tools, air, and drying halls (Lu et al., 2022; Wang, 2022). Our results indicated that the microorganisms introduced via the raw sorghum may also contribute to the brewing microbiota. This may be because most of the processes, such as grain wetting, steaming, stacking, and cellaring, are conducted in the same undivided workshop during the production. Before grain wetting, workers spread several sorghum grains out of their packages and stack them into piles in the workshop as they wait for water to be added, allowing the microorganisms present on the sorghum surface to be dispersed into the surrounding environment. These microorganisms adhere to the drying halls, cellars, and tools during subsequent stacking and fermentation sessions, which initiates inoculation of the fermented grains.

The special flavor of Jiang-flavored baijiu is formed by the metabolites of different microorganisms that use fermented grains as the culture medium. Therefore, predicting the metabolic functions of microorganisms and analyzing the correlation between the microorganisms and the flavor substances of the baijiu play key roles in maintaining product quality (Tan et al., 2022). In our study, we found a correlation between ethyl acetate, ethyl caprylate, ethyl lactate, butyl caproate, and the microorganisms on the surface of raw materials in the first batch of baijiu. These results enrich the theory of the correlation between flavor substances and the microbiota of Jiang-flavored baijiu and provide a reference basis for further research.

Using functional gene prediction, we found that Proteobacteria, Bacteroidota, Firmicutes, Acidobacteriota, Actinobacteriota, Chloroflexi, Myxococcota, and Gemmatimonadota were strongly correlated with metabolic pathways for carbohydrates, amino acids, energy and cofactors, and vitamins. Acetobacter, Bacillus, and Lactobacillus species within the phyla Proteobacteria and Firmicutes were identified as key contributors to the production of ethyl lactate, butyric acid, hexanoic acid, lactic acid, and pyrazine compounds in baijiu. Bacillus species can break down proteins and starches into amino acids and monosaccharides via proteases and amylases, as well as generate organic acids via the tricarboxylic acid cycle. These microorganisms play crucial roles in the fermentation process of baijiu production (Wang et al., 2016; Pan et al., 2023). Although the abundance of Bacillus was not high at the genus level in this study, it does not exclude the possibility that microbial community succession may have made it the dominant species in the subsequent brewing process due to environmental changes. Finally, the number of functional genes in the bacterial communities of the samples varied slightly but not significantly, suggesting that there are other factors that determine the functional changes in the brewing microbial community besides the surface microorganisms of the raw materials, which have only a partial functional impact.

The quality of sorghum crops and the composition of surface microbiota are influenced by a variety of factors, including crop variety, cultivation techniques, ecological and climatic conditions, and soil characteristics (Bokulich et al., 2014; Ding et al., 2021; Chen et al., 2023). Guizhou is a karst farmland ecosystem characterized by intricate geomorphology and significant microclimate variations (Wang et al., 2019). Variations in soil composition can affect the development and spatial extent of sorghum root systems, affecting the ability of plants to absorb water and nutrients (Zhao and Hou, 2019). Climate variations affect sorghum photosynthesis and influence the rate of photosynthetic product accumulation (Zegada-Lizarazu et al., 2015). The interaction between the soil and climate influences nutrient transport and grain accumulation, leading to variations in sorghum quality. Additionally, alterations in topography, climate, and soil conditions modify the composition and structure of the sorghum microbiota by affecting the hydrothermal conditions in the field (Khalifa and Eltahir, 2023). In our study, we found that Pseudomonas, Pantoea, and Kosakonia were significantly correlated with soil total phosphorus, elevation and diurnal temperature difference, while the abundance of Bacteroides, unclassified Vicinamibacterales and Sphingomonas was mainly affected by the monthly mean temperature, precipitation, and effective accumulative temperature.

Although investigations into the surface microorganisms of sorghum are lacking, recent studies have explored the surface microorganisms of grapes as key components in wine production. Proteobacteria and Ascomycota were identified as the predominant bacterial and fungal taxa, respectively, in the microbial communities present on the surfaces of grapes in Xinjiang (Gao et al., 2019). The diversity of bacterial communities was associated with altitude, latitude, and longitude; whereas, the fungal community diversity was significantly influenced by the altitude, dryness, frost-free period, latitude, and longitude. By contrast, the microbial composition on grape surfaces in the Grenache and Carignan regions of Spain was characterized by the dominance of Ascomycetes, followed by Actinobacteria, Acidobacteria, and Bacillus, which was a significant factor in the distribution of bacteria (Portillo et al., 2016). Previous studies have successfully established correlations between the microbiota of raw brewing materials and ecological factors, albeit without consideration of varietal differences. To address this gap, we used a consistent variety of waxy sorghum to eliminate varietal influence. Our findings indicated that the microbiological composition of sorghum surfaces was influenced by a combination of the climate, altitude, and soil properties. However, this study only covers a relatively small area of the country and did not address how the microbial composition may also be influenced by land-use history or other bioenvironmental factors.

## 5 Conclusion

Variations in the physicochemical characteristics and surface microbial community diversity were observed in Hongliangfeng No. 1 sorghum cultivated in the RH, DY, and JS regions. The results showed that the difference of origin not only affected total starch, amylose, protein, and tannin content, but also formed a unique composition of surface microorganisms at different phylum and genus levels, with both the local soil and climate can have a key impact on the sorghum bacterial community. There was a correlation between the microbial diversity and baijiu flavor components, which provides a reference for further exploration of the brewing function of the raw sorghum materials. Our future research will further expand the range of sample collection, including after wetting, analyzing the brewing environment (e.g., tools, air, plant, and ground) during the same period and collecting data on additional environmental factors to help clearly establish the sources and metabolic pathways of microorganisms found on the surface of waxy sorghum and how they affect the flavor of Jiang-flavored baijiu.

## Data availability statement

The original contributions presented in the study are publicly available. This data can be found in the Genome Sequence Archive (Genomics, Proteomics & Bioinformatics 2021) in National Genomics Data Center (Nucleic Acids Res 2022), China National Center for Bioinformation/Beijing Institute of Genomics, Chinese Academy of Sciences (GSA: CRA CRA017410) that are publicly accessible at https://ngdc.cncb.ac.cn/gsa.

## Author contributions

PX: Writing – review & editing, Writing – original draft, Investigation, Conceptualization. MS: Writing – original draft, Supervision, Methodology. XD: Writing – review & editing, Supervision. YR: Writing – review & editing, Validation. MC: Writing – review & editing, Software, Investigation. YJ: Writing – review & editing, Investigation. JS: Writing – review & editing, Writing – original draft, Conceptualization.

## Footnotes

^1^ https://www.originlab.com/2021

^2^ http://www.canoco5.com/
