# Supplementary material for: Bacterial composition and physicochemical characteristics of sorghum based on environmental factors in different regions of China
Source: Front Microbiol. 2024 Jun 28;15:1422471. doi: 10.3389/fmicb.2024.1422471 (PMC11240854; doi:10.3389/fmicb.2024.1422471)
Supplement: Supplementary file 1 [file Data_Sheet_1.docx]

**Appendice**

S1 Contents of chemical components of sorghum grains

| Area | Total starch content/% | Amylose content/% | Amylopect content/% | Lipids content/% | Protein content/% | Tannin content/% |
| --- | --- | --- | --- | --- | --- | --- |
| JS | 66.53±0.04a | 5.38±0.02b | 66.15±0.09a | 4.12±0.11b | 13.43±0.99a | 1.01±0.04b |
| RH | 64.94±0.25b | 3.06±0.13b | 61.88±0.10a | 4.32±0.11b | 12.56±0.13a | 1.41±0.04a |
| DY | 65.32±0.08ab | 10.86±0.02a | 54.46±0.06b | 6.93±0.12a | 11.16±0.14a | 1.42±0.03a |


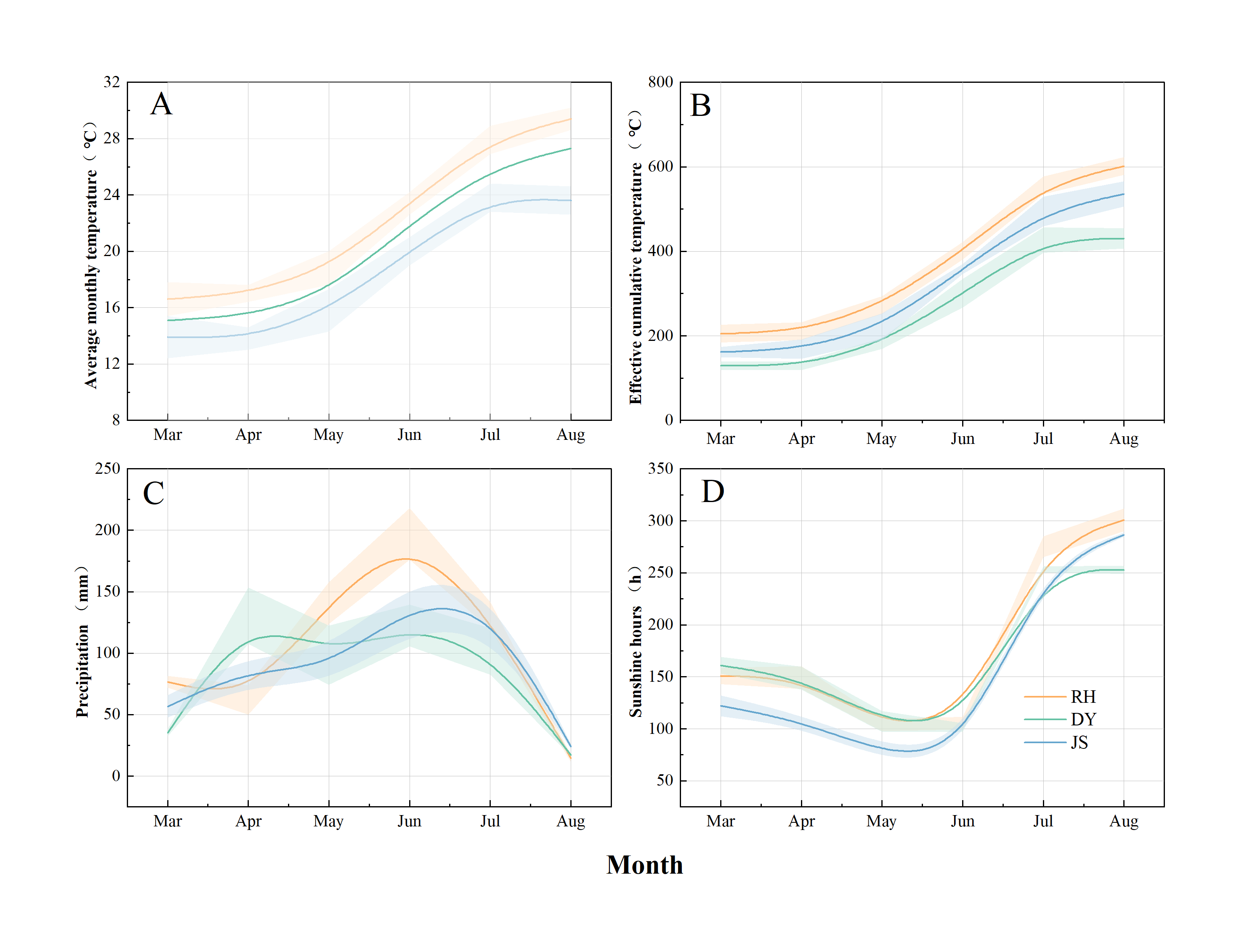
S2 Climate change characteristics of different production areas in 2022


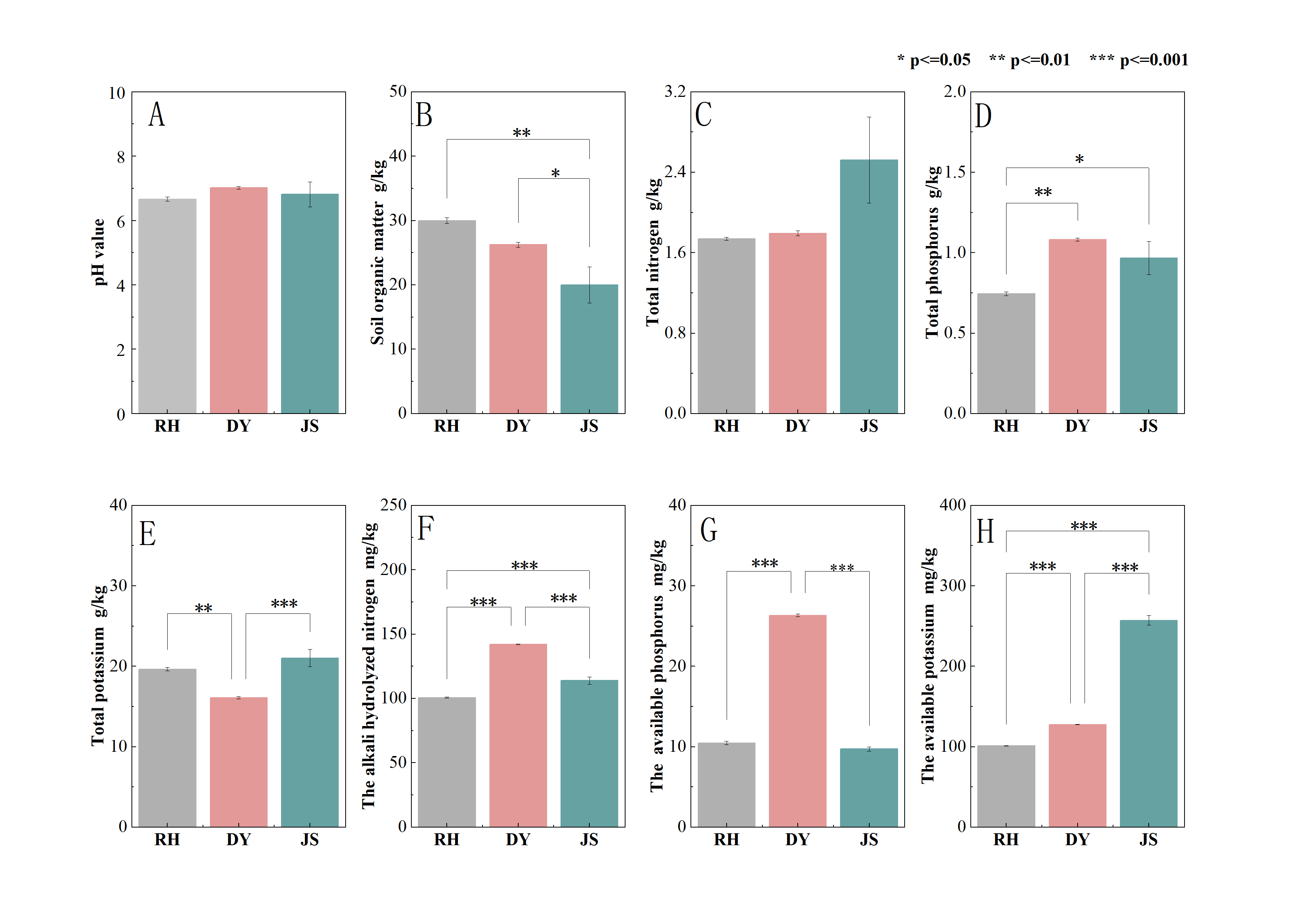


S3 Characteristics of soil elements in different production areas

S4  KEGG Database Level 1 Functional Classification Statistics

| No. | Function Types | JS1 | JS2 | JS3 | RH1 | RH2 | RH3 | DY1 | DY2 | DY3 | SUM |
| --- | --- | --- | --- | --- | --- | --- | --- | --- | --- | --- | --- |
| 1 | Metabolism | 73765700 | 83060878 | 96956577 | 159391688 | 74768043 | 65631327 | 107768785 | 86530971 | 101679156 | 849553125 |
| 2 | Genetic Information Processing | 7049578 | 6980061 | 9246076 | 11758855 | 7098065 | 6239023 | 10240121 | 8246249 | 9643655 | 76501683 |
| 3 | Environmental Information Processing | 5694598 | 8741300 | 7554940 | 17959030 | 5835875 | 5132686 | 8417262 | 6695202 | 7969186 | 74000079 |
| 4 | Human Diseases | 2811767 | 3459589 | 3849400 | 6768699 | 2965551 | 2594127 | 4277689 | 3392157 | 4001280 | 34120259 |
| 5 | Cellular Processes | 2372662 | 3453275 | 2687454 | 7616368 | 2131669 | 1896464 | 3085038 | 2510395 | 2957287 | 28710612 |
| 6 | Organismal System | 1748634 | 1829620 | 2540195 | 3465636 | 1942158 | 1688870 | 2794096 | 2201124 | 2597435 | 20807768 |

| No. | Metabolic pathways | JS1 | JS2 | JS3 | RH1 | RH2 | RH3 | DY1 | DY2 | DY3 | SUM |
| --- | --- | --- | --- | --- | --- | --- | --- | --- | --- | --- | --- |
| 1 | Global and overview maps | 40062264 | 44686966 | 52880559 | 84503862 | 40738533 | 35749292 | 58719858 | 47128924 | 55344293 | 459814551 |
| 2 | Carbohydrate metabolism | 7334679 | 9200147 | 9104059 | 16376747 | 7068083 | 6250934 | 10206944 | 8274439 | 9818164 | 83634196 |
| 3 | Amino acid metabolism | 5951085 | 6830446 | 7377018 | 16215787 | 5743917 | 5059639 | 8285069 | 6721103 | 7845752 | 70029816 |
| 4 | Energy metabolism | 5609382 | 5563267 | 8186843 | 9093828 | 6233738 | 5419081 | 8965989 | 7069229 | 8306925 | 64448282 |
| 5 | Metabolism of cofactors and vitamins | 4673988 | 4876821 | 6549053 | 8618540 | 5006220 | 4368658 | 7210064 | 5730823 | 6725819 | 53759986 |
| 6 | Nucleotide metabolism | 3124563 | 3435289 | 4128633 | 5720098 | 3173253 | 2789021 | 4573332 | 3672847 | 4319348 | 34936384 |
| 7 | Metabolism of other amino | 1509826 | 1839190 | 2063976 | 3555303 | 1588155 | 1389729 | 2285932 | 1819160 | 2142935 | 18194206 |
| 8 | Lipid metabolism | 1565106 | 1864767 | 1789423 | 4433653 | 1411639 | 1254789 | 2035181 | 1670830 | 1951913 | 17977301 |
| 9 | Xenobiotics biodegradation and metabolism | 1029508 | 1534301 | 1233052 | 5064572 | 984767 | 865557 | 1414805 | 1141941 | 1332603 | 14601106 |
| 10 | Glycan biosynthesis and metabolism | 995203 | 1158082 | 1203040 | 1872688 | 926361 | 823324 | 1344136 | 1104631 | 1303379 | 10730844 |
| 11 | Metabolism of terpenoids and polyketides | 787895 | 865241 | 1053771 | 1548483 | 813962 | 711439 | 1169168 | 934497 | 1095114 | 8979570 |
| 12 | Biosynthesis of secondary metabolites | 806208 | 868844 | 1014096 | 1471521 | 786576 | 690952 | 1135849 | 917265 | 1094271 | 8785582 |

S5 KEGG Database Level 2 Functional Classification Statistics
